# Paleovirology—Modern Consequences of Ancient Viruses

**DOI:** 10.1371/journal.pbio.1000301

**Published:** 2010-02-09

**Authors:** Michael Emerman, Harmit S. Malik

**Affiliations:** 1 Division of Human Biology, Fred Hutchinson Cancer Research Center, Seattle Washington, United States of America; 2 Division of Basic Sciences, Fred Hutchinson Cancer Research Center, Seattle Washington, United States of America; 3 Howard Hughes Medical Institute, Fred Hutchinson Cancer Research Center, Seattle Washington, United States of America; Washington University School of Medicine, United States of America

Within the past century, a number of “emerging viruses” with pathogenic properties, such as HIV-1, SARS-CoV, and several novel reassortments of influenza A, have entered the human population on a large scale. However, novel pathogenic viral infections of humans are not unique to modern history. “Paleovirology” is the study of ancient extinct viruses (called “paleoviruses”) and the effects that these agents have had on the evolution of their hosts. Thus far, the study of these viruses has mostly been limited to endogenous retroviruses that can be directly identified from their remnants in host genomes. However, one can infer the existence of other paleoviruses from their evolutionary pressures on host genes. We suggest that selection to survive the pathogenic effects of these viruses has shaped our repertoire of antiviral defenses in ways that impact our resistance or susceptibility to modern-day emerging viruses.

## Unearthing Signs of Ancient Viral Infections

**Figure 1:**
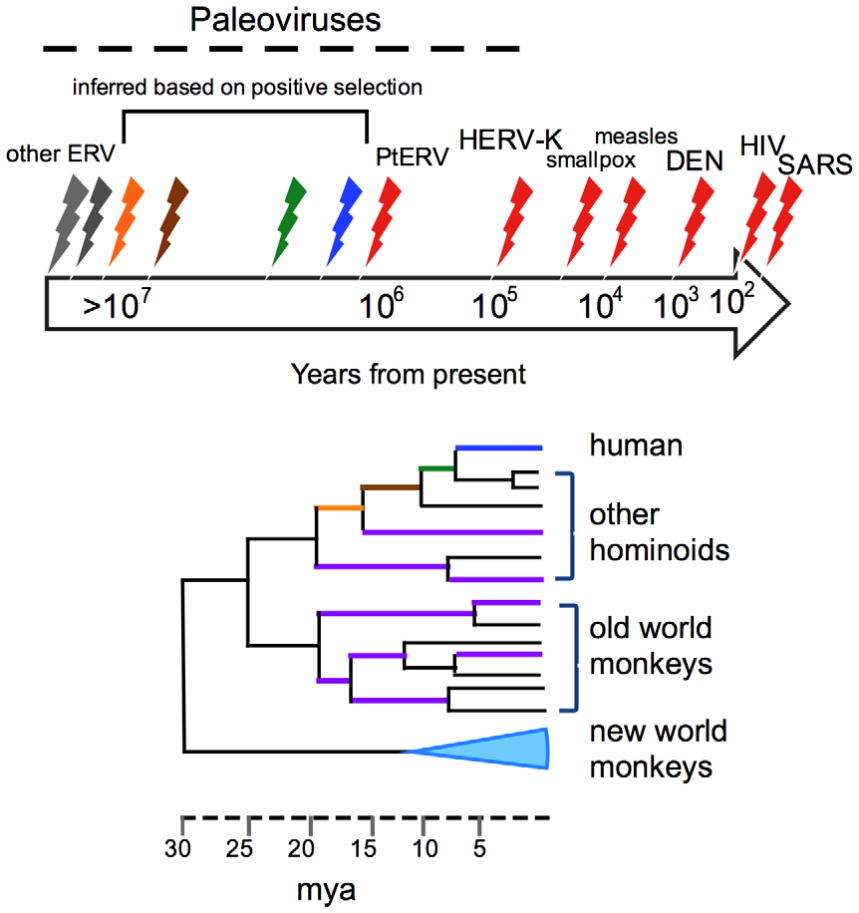
Time-line of paleoviruses in the human lineage. The dashed line at the top line represents the time period for paleoviruses. The red lightning bolts represent dates of known recent and ancient viruses based either on historical records, molecular clocks, or on endogenous retroviruses in the human genome for SARS-Co [38], HIV-1 and -2 [39], dengue (DEN) [40], measles [13], smallpox [41], HERV-K(HML2) [42], or PtERV [27], and older endogenous retroviruses shared among all hominoids or all primates [43]. The blue, green, and orange lightning bolts represent inferred viruses based on positive selection of TRIM5 [18], and the brown line is an inferred virus based on positive selection of ZAP [19] simplified for representation, here. Each color corresponds to inferred paleoviruses based on positive selection on a particular antiviral gene calculated on the phylogenetic tree in the bottom of the figure where the lineage under selection has the same color coding, and the dates correspond to dates of the ancestors [44]. Although one virus is shown per episode of selection, there could be many different waves of similar viruses during that time period. Purple branches refer to selections due to inferred paleoviruses in lineages that do not lead directly to humans. There is considerable uncertainty associated with most of the dates referred to in this figure.

The human genome is a living document of ancient and now extinct viruses. Indeed, DNA of retroviral origin makes up 8% of human genome sequence. Retroviruses are RNA viruses that replicate through a DNA intermediate called a provirus, which becomes integrated into the host cell chromosome. Usually such integrations occur in somatic cells, but when integration of the provirus occurs in a germ cell, an endogenous retrovirus can be inherited as part of the genome. If these germline insertions become fixed within a population, the provirus becomes part of the genetic legacy of the species. It is difficult to calculate exactly how many retroviral infections of the germline led to the ∼100,000 copies of endogenous retroviruses in the human genome because duplications, transpositions, and other non-infectious events also contribute to this number. However, at a very minimum, each of the more than 31 families of endogenous retrovirus found in the human genome [1] must have arisen from one or more separate paleoviruses that infected the ancestors of modern humans. Since reinfections of the germline with members of the same families occurred frequently [2], retroviral infections that impacted the genome had to have happened repeatedly during primate evolution with the most recent episode in humans between 100,000 and 1 million years ago [3] (Figure 1).

This impressive fossil record, represented by endogenous retroviruses, is still likely a vast underestimate of the number of retroviral infections of human ancestors. Many retroviruses do not infect the germline. For example, the human T cell leukemia virus types I and II (HTLVI and II) are thought to have entered human populations over 20,000 years ago [4], but no endogenous copies in human genomes have yet been found. For those retroviruses that do infect the germline, the majority of integration events did not become fixed since they were negatively selected out of the population or lost by drift. Indeed, the integration pattern of endogenous retroviruses (usually located away from genes) is quite different from the integration pattern of their exogenous counterparts (often located near or in genes) [5],[6], suggesting that selection has cleansed the majority of endogenization events from primate genomes. These imperfections in the fossil record of endogenous retroviruses can lead to vast underestimation of the age of viral lineages, as was recently demonstrated for the lentiviruses (retroviruses such as HIV-1) where the finding of endogenized copies in two independent lemur genera [7],[8] upwardly revised the age of primate lentiviruses to more than 4 million years.

It has been challenging for paleovirology to move beyond the study of endogenous retroviruses since it is much more difficult to decipher ancient viruses that left no extant copies of their past existence. The recent finding that at least one bornavirus gene has integrated in several mammalian genomes at multiple evolutionary periods demonstrates both the possibility of identifying and dating some other ancient classes of viral infections [9] although this remains a rare event outside of the retrovirus family. Nonetheless, endogenous copies of viruses are but a subset of the ancient and extinct viruses that we call paleoviruses. The existence of some additional paleoviruses can be deciphered by estimating the age of the last common ancestor of extant viruses. For viruses where there is evidence for co-speciation of virus and host, we can arrive at a conservative estimate of their evolutionary age by correlation to the divergence of their hosts. The best example of primate viruses that have cospeciated with its host over a considerable evolutionary period are the spumaretroviruses [10]. For other viruses, like herpesviruses and papillomaviruses, there is also some support for host–virus cospeciation [11],[12].

In contrast, DNA viruses such as orthopoxviruses show ample evidence of host switching and have no suitable cospeciation estimates. In such cases, although we can calculate the time to the last common ancestor from estimates of mutation rates in extant sequences, this is likely to be an underestimate due to the extinction of viral lineages (and thus, the loss of their sequence diversity in the calculation) [13]. Moreover, these age estimates do not provide any information as to which host lineages might have been affected by any given inferred paleovirus. However, tracing the acquisitions of genes and retroelements from host genomes into large DNA viruses may provide an additional means to estimate their divergence times [14]–[16]. On the other hand, the case for ancient RNA viruses, which provide more examples of pathogenic viruses than DNA viruses, is even more vexing because high mutation rate essentially obliterates bioinformatic signals and our ability to meaningfully estimate ancestry beyond a few million years [13]. Indeed, based on mutation rates and sampled lineages, one might incorrectly infer that the last common ancestor for most RNA viruses was very young [13].

## Finding an Evolutionary Signal of Ancient Infections within Antiviral Genes

Given these problems in using the sequences of current day viruses to identify most paleoviruses, we propose another approach to infer the existence of pathogenic paleoviruses. This approach is based on a striking evolutionary signature called positive selection that these viruses impart on antiviral genes in primate genomes. When a viral infection rages in a population, pre-existing variants of these antiviral defense genes are acted upon by Darwinian selection, leading to the accelerated fixation of even previously rare variants in the species. It is important to emphasize that the inability to ward off a viral infection can translate to significant fitness costs. Therefore, beneficial variants in antiviral genes will spread to fixation by virtue of their significant selective advantage even if viral infection does not lead to death. Counterevolution by the virus (or introduction of new viruses that evade the host defenses using the same mechanisms) eventually leads to renewed selective pressure on antiviral genes. These recurrent bouts of selection represent evolutionary arms races that can be detected by comparing DNA coding sequences of related species and by using maximum likelihood methods looking for excesses of mutations that change amino acids in gene sequences (non-synonymous mutations) relative to mutations that do not change amino acids (synonymous mutations) [17]. Repeated episodes of these arms races between host antiviral genes and new viral challenges will lead to dramatic rates of change of non-synonymous mutations. Thus, signatures of evolutionary changes in protein-coding sequences of antiviral genes allow us to infer the selection of ancient host species due to paleoviruses.

Of course, it would be incorrect to imply that the complete outcome of a viral infection is decided on the basis of a single host antiviral gene. If this were true, then the differences in tempo of viral versus host evolution would make it unlikely that the hosts could ever adapt away from a viral infection (Box 1). Instead, the host–virus interaction is more complex, where alleles in single host genes vary in relative resistance/susceptibility to viruses. The outcome of a particular viral infection on a population level is determined by the compendium of antiviral genes borne by the host, the viral fitness cost associated with escape from antiviral genes, as well as many factors beyond host and viral genetics. Nonetheless, over evolutionary timescales, the effects of single genes can be discerned as long as beneficial alleles confer fitness advantages to the host.

**Box 1: Problems in Paleovirology**

What ancient viruses have caused selective pressure along the human lineage?

Is it possible to correlate evidence for ancient viruses with any other major transitional events in the evolutionary history of animals or plants?

Do some viruses that were once eliminated from populations of human ancestors have ecological reservoirs with the potential to be re-introduced into humans?

How do the different replication rates/mutation frequencies between viruses and their hosts affect the population-level dynamics and evolutionary signatures of genetic conflict on host genes?

What are the consequences of natural selections due to ancient viruses, or relaxed selection due to loss of pathogenic pressure, on modern viral diseases?

Do certain types of antiviral genes show correlated positive selection along certain branches of the primate phylogeny, suggesting their action against a common pathogen?

The comparison of sequence data from multiple orthologs of primate antiviral genes allows us to not only infer that a given gene was under positive selection, but also when it was under positive selection. This can be done by reconstructing the ancestral coding sequence of an antiviral gene at each node of a primate phylogenetic tree in silico and then determining where positive selection occurred on internal nodes (the internal nodes can be dated by other molecular evolutionary methods, assuming some sort of molecular clock for mutation rates in primate genomes, or by paleontological fossil records). Each case of positive selection for an antiviral gene on the phylogenetic tree then implies the presence of at least one paleovirus on that branch (Figure 1). For instance, since diverging from the chimpanzee lineage, human TRIM5, a potent antiviral factor, has incurred eight non-synonymous and two synonymous changes—this roughly translates to an excess of three non-synonymous changes over what might be expected by chance [18]. This information suggests the presence of at least one paleovirus that was in genetic conflict with TRIM5 in the 4–5 million years in the human lineage since the human–chimpanzee divergence (Figure 1). However, there is no information about when each of these three changes became fixed in the population—this could have occurred in rapid succession or slowly over 4–5 million years. When the positive selection of different unlinked antiviral genes are compared to one another [18]–[21], one finds different patterns of episodic positive selection in primate evolution for each gene. This suggests that different ancient viral pathogens acting at different times were responsible for driving selection of one gene versus another. In theory, episodes of positive selection could also occur on multiple antiviral genes due to the same pathogenic virus (Box 1) if the fitness costs imposed by a particular virus were especially severe at any given period of primate evolution.

Another means to date a paleoviral infection is the finding of a gene fusion event in a particular primate lineage that resulted in novel antiviral activity, exemplified by the TRIM5 fusion to a CypA retrogene in the *Aotus* genus of New World monkeys [22],[23]. Dating the origin of such a gene fusion can be informative even if the “newly born” antiviral gene eventually degrades due to relaxed selection (once the paleoviral challenge is extinguished). In both cases (positive selection or gene fusions), the time-period under selection can only be ascribed to a branch on the evolutionary tree, which may represent several million years of evolution (the age represented by the branch is determined by the phylogenetic density of extant species and extent of sequence sampling). Other methods can examine the positive selection on antiviral genes in shorter time-scales by looking for evidence of selective sweeps using single nucleotide polymorphisms in modern population data, for instance in human populations [24],[25]. These methods are especially useful in determining the action of a relatively “modern” selective pathogen which may or may not also have had an ancient counterpart. Finding both modern and ancient selective episodes is strong indication of a particular antiviral gene having been repeatedly acted upon by antagonism against both ancient paleoviruses and present-day viral pathogens.

## Challenges and Opportunities in Using Evolution to Infer Paleovirology

There are limitations to inferring episodes of paleoviral infection from signatures of positive selection. Some weaknesses in the method arise directly from methodology; by definition, it is difficult, if not impossible, to detect a single episode of positive selection if it occurred in the distant past. Thus, for the most part, we are left considering only those antiviral genes that have been repeated targets of selection. Moreover, our ability to use reconstructive evolution to infer positive selection may weaken at deep evolutionary distances, although the 35-million-year age of simian primate evolution is especially tractable. An additional limitation is the fact that the same antiviral gene may have acted on different viruses over the evolutionary record, with each new episode potentially “over-writing” the previous one. Each instance of adaptation may involve only a few amino acid changes, but if these occur at the same position then the record can get muddled in terms of elucidating exactly which change was selected when. This limitation is exemplified by the human major histocompatibility (MHC) class I genes devoted to the presentation of viral antigens to the adaptive immune system. The vast diversity and rapid evolution of alleles in MHC Class I demonstrates that humans and primate ancestors have undergone dramatic episodes of positive selection to protect against continual and diverse viral attack [26]; however the constant turnover has eroded information about all but the most recently encountered viruses. Finally, the evolutionary analysis of antiviral genes that also play a role in limiting other microbial pathogens would confound the assessment of what type of pathogen was responsible for the positive selection.

**Figure 2:**
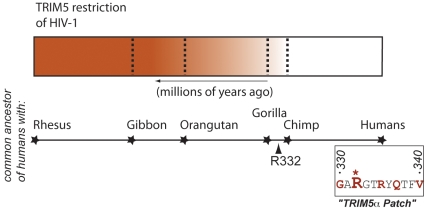
TRIM5 restriction of HIV-1 has decreased during evolution leading to humans. The shading of the rectangle represents the degree that TRIM5 will limit infection of HIV-1 (darker color means TRIM5 decreases HIV-1 infection more) and the X-axis indicates time in millions of years from the present. Each dotted line represents the reconstruction of TRIM5 as it likely existed at a node of a phylogenetic tree indicating a common ancestor of humans with chimpanzees, gorillas, orangutans, gibbons, and Old World monkeys (rhesus). Original data is found in [30] and shows that the antiviral gene TRIM5 restricted HIV-1 better at points in evolution earlier than the chimp–human common ancestor than it does after that. On the right shows an amino acid sequence of a region of TRIM5 containing amino acids that confer resistance or susceptibility to HIV-1 with the amino acids that are under the strongest positive selection in red [14]. Changes in this region cause a gain of restriction to some viruses, while causing a loss to others [33],[45]–[47]. The R332 amino acid, which represents the single largest determinant of loss of resistance to HIV-1 [48],[49], was fixed before the chimp–human common ancestor, but positive selection has continued in TRIM5 along the human lineage beyond this point.

Notwithstanding these caveats, we believe it is possible to identify candidate classes of paleoviruses based on analyses of positive selection in antiviral genes (Box 1). First, our knowledge of the functions of antiviral genes under selection provides clues to which viruses might be considered. For instance, some antiviral genes are only active against a limited range of viral lineages. Thus, ancient episodes of positive selection in TRIM5 are likely indicative of ancient retroviral infections where the evolutionary loss of the ability of TRIM5 to combat HIV shows that ancient selective events that changed the specificity of TRIM5 clearly have had a detrimental effect on the resistance of modern humans to this contemporary virus (Figure 2). Moreover, even when the antiviral gene is embattled with several viruses simultaneously, the amino acid positions that have evolved under positive selection can be a good indication of what kind of antagonism was encountered during a given evolutionary period. For example, even through protein kinase R can act as a general antiviral protein, evolutionary reconstructions have allowed us to infer the presence of virus-encoded mimics of eIF2alpha throughout the history of primate evolution, despite the fact that such mimics are known in only a few extant and relatively young lineages of poxviruses and iridoviruses [21].

Second, since the rapid evolution of host antiviral genes is likely driven by their genetic conflict with specific viral antagonists, the classification of which modern viruses can neutralize the function of which antiviral genes under positive selection can help define classes of possible candidate ancient viral antagonists and thereby pathogens. Similarly, there may be cases of “missing” viral species where extinction of a virus that is present in sister primate species could indicate the aftermath of a virus–host interaction that was beneficial to one host lineage. Examples include the PtERV retrovirus that is not present in human genomes but is present in chimpanzee and gorilla genomes [27], a lineage of rhadinovirus that has not yet been found in humans but is present in sister taxa [28], and foamy viruses that are present in most primates but not humans [29]. Finding a human gene active against a virus that is not found in humans could be a starting point for considering whether or not an older version of that virus was eliminated from the evolutionary lineage leading to modern humans due to genetic adaptation rather than geographical isolation.

## What Paleovirology Can Tell Us

Why study pleovirology? Paleovirology could be viewed as the study of ancient viruses that primate genomes encountered and defeated during the course of evolution. This view emphasizes that our current antiviral repertoire was not optimized to combat present infections, but rather is the product of selection for survival of our species' past infections. Thus, the selective changes that these antiviral genes incurred during these periods of evolutionary pressure might make them less competent to fight modern viral challenges (Figure 2; Box 1). For example, the human TRIM5 gene does not inhibit HIV, although it was certainly selected to inhibit something else in our past [30]. The analysis of amino acids on antiviral genes driven by selection of ancient pathogens can be used to identify the interface between the host protein and the virus (Figure 2) in ways that could conceivably be used to design rational antiviral drugs or gene therapy strategies. Such analyses of the virus–host battles, on an evolutionary scale, can also explain the otherwise mysterious loss of antiviral activities. For instance, antiviral genes that serve no other cellular functions can incur significant fitness costs or relaxed selection and therefore can be lost due to the lack of a pathogen during extended periods of time [20],[31]. These changes and losses to our antiviral repertoire may help explain deficiencies in our current innate immune protection against pathogenic viral challenges.

The study of “resurrected” paleoviruses, exemplified by the evolution-guided reconstruction of several endogenous retroviruses and the 1918 influenza virus [32]–[36] can also reveal previously hidden details of host–virus interactions. Finally, the universe of possible “emerging viruses” that could arise by cross-species transmission of viruses from other animals into humans [37] is still largely undefined. It is possible that amidst this reservoir of potential pathogens lie the descendants of viruses that were once eliminated from human ancestors, but because of continued evolution of both virus and humans could now reinfect modern humans. Although we have focused on paleoviruses of humans and primate ancestors in this essay, the same arguments can be made and lines of research can be applied to nearly any set of animal or plant families for which virus–host relationships exist. Paleovirology, in this broader sense, may be able to correlate the existence of ancient infections with known phylogeographical events, such as extinctions, bursts of speciation, exchanges of fauna between continents, island isolations, and population migrations (Box 1). We look forward to further community efforts to identify candidate paleoviruses, analyze the evolutionary signatures of suspected paleoviral infections, and determine how these ancient evolutionary battles have affected our ability to combat new and recurrent viral diseases of humans.

## References

[1] Katzourakis A, Rambaut A, Pybus O. G (2005). The evolutionary dynamics of endogenous retroviruses.. Trends Microbiol.

[2] Belshaw R, Pereira V, Katzourakis A, Talbot G, Paces J (2004). Long-term reinfection of the human genome by endogenous retroviruses.. Proc Natl Acad Sci U S A.

[3] Bannert N, Kurth R (2006). The evolutionary dynamics of human endogenous retroviral families.. Annu Rev Genomics Hum Genet.

[4] Salemi M, Desmyter J, Vandamme A. M (2000). Tempo and mode of human and simian T-lymphotropic virus (HTLV/STLV) evolution revealed by analyses of full-genome sequences.. Mol Biol Evol.

[5] Brady T, Lee Y. N, Ronen K, Malani N, Berry C. C (2009). Integration target site selection by a resurrected human endogenous retrovirus.. Genes Dev.

[6] Medstrand P, van de Lagemaat L. N, Mager D. L (2002). Retroelement distributions in the human genome: variations associated with age and proximity to genes.. Genome Res.

[7] Gifford R. J, Katzourakis A, Tristem M, Pybus O. G, Winters M (2008). A transitional endogenous lentivirus from the genome of a basal primate and implications for lentivirus evolution.. Proc Natl Acad Sci U S A.

[8] Gilbert C, Maxfield D. G, Goodman S. M, Feschotte C (2009). Parallel germline infiltration of a lentivirus in two Malagasy lemurs.. PLoS Genet.

[9] Horie M, Honda T, Suzuki Y, Kobayashi Y, Daito T (2010). Endogenous non-retroviral RNA virus elements in mammalian genomes.. Nature.

[10] Switzer W. M, Salemi M, Shanmugam V, Gao F, Cong M. E (2005). Ancient co-speciation of simian foamy viruses and primates.. Nature.

[11] Gottschling M, Stamatakis A, Nindl I, Stockfleth E, Alonso A (2007). Multiple evolutionary mechanisms drive papillomavirus diversification.. Mol Biol Evol.

[12] Sharp P. M (2002). Origins of human virus diversity.. Cell.

[13] Holmes E. C (2008). Evolutionary history and phylogeography of human viruses.. Annu Rev Microbiol.

[14] Hughes A. L, Friedman R (2005). Poxvirus genome evolution by gene gain and loss.. Mol Phylogenet Evol.

[15] Piskurek O, Okada N (2007). Poxviruses as possible vectors for horizontal transfer of retroposons from reptiles to mammals.. Proc Natl Acad Sci U S A.

[16] Wang N, Baldi P. F, Gaut B. S (2007). Phylogenetic analysis, genome evolution and the rate of gene gain in the Herpesviridae.. Mol Phylogenet Evol.

[17] Holmes E. C (2004). Adaptation and immunity.. PLoS Biol.

[18] Sawyer S. L, Wu L. I, Emerman M, Malik H. S (2005). Positive selection of primate TRIM5alpha identifies a critical species-specific retroviral restriction domain.. Proc Natl Acad Sci U S A.

[19] Kerns J. A, Emerman M, Malik H. S (2008). Positive selection and increased antiviral activity associated with the PARP-containing isoform of human zinc-finger antiviral protein.. PLoS Genet.

[20] OhAinle M, Kerns J. A, Li M. M, Malik H. S, Emerman M (2008). Antiretroelement activity of APOBEC3H was lost twice in recent human evolution.. Cell Host Microbe.

[21] Elde N. C, Child S. J, Geballe A. P, Malik H. S (2009). Protein kinase R reveals an evolutionary model for defeating viral mimicry.. Nature.

[22] Ribeiro I. P, Menezes A. N, Moreira M. A, Bonvicino C. R, Seuanez H. N (2005). Evolution of cyclophilin A and TRIMCyp retrotransposition in New World primates.. J Virol.

[23] Sayah D. M, Sokolskaja E, Berthoux L, Luban J (2004). Cyclophilin A retrotransposition into TRIM5 explains owl monkey resistance to HIV-1.. Nature.

[24] Sabeti P. C, Schaffner S. F, Fry B, Lohmueller J, Varilly P (2006). Positive natural selection in the human lineage.. Science.

[25] Sabeti P. C, Varilly P, Fry B, Lohmueller J, Hostetter E (2007). Genome-wide detection and characterization of positive selection in human populations.. Nature.

[26] Worobey M, Bjork A, Wertheim J. O (2007). Point, counterpoint: The evolution of pathogenic viruses and their human hosts.. Annual Review of Ecology Evolution and Systematics.

[27] Yohn C. T, Jiang Z, McGrath S. D, Hayden K. E, Khaitovich P (2005). Lineage-specific expansions of retroviral insertions within the genomes of African great apes but not humans and orangutans.. PLoS Biol.

[28] Lacoste V, Mauclere P, Dubreuil G, Lewis J, Georges-Courbot M. C (2001). A novel gamma 2-herpesvirus of the Rhadinovirus 2 lineage in chimpanzees.. Genome Res.

[29] Linial M, Knipe D, Howley P. M (2007). Foamy Viruses.. Fields Virology. 5th ed.

[30] Goldschmidt V, Ciuffi A, Ortiz M, Brawand D, Munoz M (2008). Antiretroviral activity of ancestral TRIM5alpha.. J Virol.

[31] Venkataraman N, Cole A. L, Ruchala P, Waring A. J, Lehrer R. I (2009). Reawakening retrocyclins: ancestral human defensins active against HIV-1.. PLoS Biol.

[32] Dewannieux M, Harper F, Richaud A, Letzelter C, Ribet D (2006). Identification of an infectious progenitor for the multiple-copy HERV-K human endogenous retroelements.. Genome Res.

[33] Kaiser S. M, Malik H. S, Emerman M (2007). Restriction of an extinct retrovirus by the human TRIM5alpha antiviral protein.. Science.

[34] Lee Y. N, Bieniasz P. D (2007). Reconstitution of an infectious human endogenous retrovirus.. PLoS Pathog.

[35] Perez-Caballero D, Soll S. J, Bieniasz P. D (2008). Evidence for restriction of ancient primate gammaretroviruses by APOBEC3 but not TRIM5alpha proteins.. PLoS Pathog.

[36] Tumpey T. M, Basler C. F, Aguilar P. V, Zeng H, Solorzano A (2005). Characterization of the reconstructed 1918 Spanish influenza pandemic virus.. Science.

[37] Wolfe N. D, Dunavan C. P, Diamond J (2007). Origins of major human infectious diseases.. Nature.

[38] Hon C. C, Lam T. Y, Shi Z. L, Drummond A. J, Yip C. W (2008). Evidence of the recombinant origin of a bat severe acute respiratory syndrome (SARS)-like coronavirus and its implications on the direct ancestor of SARS coronavirus.. J Virol.

[39] Wertheim J. O, Worobey M (2009). Dating the age of the SIV lineages that gave rise to HIV-1 and HIV-2.. PLoS Comput Biol.

[40] Holmes E. C, Twiddy S. S (2003). The origin, emergence and evolutionary genetics of dengue virus.. Infect Genet Evol.

[41] Li Y, Carroll D. S, Gardner S. N, Walsh M. C, Vitalis E. A (2007). On the origin of smallpox: correlating variola phylogenics with historical smallpox records.. Proc Natl Acad Sci U S A.

[42] Belshaw R, Dawson A. L, Woolven-Allen J, Redding J, Burt A (2005). Genomewide screening reveals high levels of insertional polymorphism in the human endogenous retrovirus family HERV-K(HML2): implications for present-day activity.. J Virol.

[43] Blikstad V, Benachenhou F, Sperber G. O, Blomberg J (2008). Evolution of human endogenous retroviral sequences: a conceptual account.. Cell Mol Life Sci.

[44] Takahata N, Satta Y (1997). Evolution of the primate lineage leading to modern humans: phylogenetic and demographic inferences from DNA sequences.. Proc Natl Acad Sci U S A.

[45] Diaz-Griffero F, Perron M, McGee-Estrada K, Hanna R, Maillard P. V (2008). A human TRIM5alpha B30.2/SPRY domain mutant gains the ability to restrict and prematurely uncoat B-tropic murine leukemia virus.. Virology.

[46] Maillard P. V, Reynard S, Serhan F, Turelli P, Trono D (2007). Interfering residues narrow the spectrum of MLV restriction by human TRIM5alpha.. PLoS Pathog.

[47] Song B, Gold B, O'Huigin C, Javanbakht H, Li X (2005). The B30.2(SPRY) domain of the retroviral restriction factor TRIM5alpha exhibits lineage-specific length and sequence variation in primates.. J Virol.

[48] Stremlau M, Perron M, Welikala S, Sodroski J (2005). Species-specific variation in the B30.2(SPRY) domain of TRIM5alpha determines the potency of human immunodeficiency virus restriction.. J Virol.

[49] Yap M. W, Nisole S, Stoye J. P (2005). A single amino acid change in the SPRY domain of human Trim5alpha leads to HIV-1 restriction.. Curr Biol.

